# Precise positioning of Au islands within mesoporous Pd–Pt nanoparticles for plasmon-enhanced methanol oxidation†

**DOI:** 10.1039/d4sc07345b

**Published:** 2025-04-09

**Authors:** Liyang Zhu, Yunqing Kang, Miharu Eguchi, Yingji Zhao, Dong Jiang, Xiaoqian Wei, Xingtao Xu, Kenta Nakagawa, Toru Asahi, Tokihiko Yokoshima, Yusuke Yamauchi

**Affiliations:** a Department of Nanoscience and Nanoengineering, Department of Applied Chemistry, and Department of Life Science and Medical Bioscience, Graduate School of Advanced Science and Engineering, Waseda University Tokyo 169-8555 Japan eguchi@waseda.jp; b Department of Materials Process Engineering, Graduate School of Engineering, Nagoya University Aichi 464-8603 Japan yokoshima.tokihiko@material.nagoya-u.ac.jp; c Marine Science and Technology College, Zhejiang Ocean University Zhoushan 316022 China; d Australian Institute for Bioengineering and Nanotechnology (AIBN), The University of Queensland Brisbane Queensland 4072 Australia y.yamauchi@uq.edu.au; e Department of Chemical and Biomolecular Engineering, Yonsei University Seoul 03722 Republic of Korea

## Abstract

Trimetallic systems have garnered considerable attention in (electro)catalysis due to the synergistic effects resulting from the combination of three different metals. However, achieving precise control over the positioning of various metals and understanding the relationship between structure and performance remains challenging. This study introduces an approach for synthesizing Pd@Pt@Au mesoporous nanoparticles (MNPs) with distinct core–shell Pd@Pt structures, featuring well-dispersed isolated Au islands on the outer shell, improving the plasmonic effect. The electrocatalytic performance of Pd@Pt@Au MNPs in the methanol oxidation reaction (MOR) is assessed under light-induced and light-independent conditions. The results indicate significantly enhanced activity compared to commercial Pt black, with catalytic activity during MOR increasing approximately 7.5-fold under light irradiation. The external placement of Au on the shell of Pd@Pt@Au MNPs provides superior plasmonic enhancement, thereby contributing to improved catalytic performance under light irradiation. This investigation sheds light on the controlled synthesis of trimetallic MNPs and their catalytic applications, underscoring the importance of precise Au positioning for optimizing performance.

## Abstract 2

Pd@Pt@Au mesoporous nanoparticles (MNPs) with uniformly distributed Au islands are synthesized *via* a simple chemical reduction method, exhibiting significantly enhanced methanol oxidation performance due to plasmonic enhancement.

## Graphical abstract

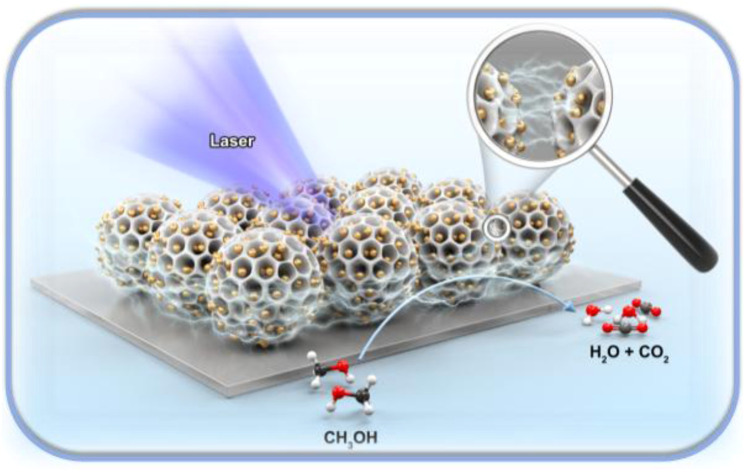

## Introduction

Trimetallic systems have become a center of innovation in the broad subject of catalysis, driving advances in a variety of applications including thermo-catalysis,^1^ photo-electrocatalysis,^2^ and electrocatalysis. These systems are powerful because they can take advantage of the special qualities of different metals, which when combined, have a synergistic impact that is greater than what can be achieved with monometallic and bimetallic systems.^3^ The precious design of trimetallic nanoparticles (NPs) enables the fine-tuning of electronic and geometric structures,^4^ unlocking unprecedented levels of catalytic activity and robustness.^5^ However, the process of precisely creating these nanoparticles is complex due to the diverse physicochemical characteristics of metals, including varying redox potentials.^6^ These differences might result in random metal distribution inside the NPs or on the surface of the NPs,^7^ unpredictable chemical reaction processes,^8^ and challenges in comprehending the structural chemistry of the NPs during intermediate growth stages.^9^ As a result, precisely adjusting the morphology of the trimetallic metals and positioning the different metals remains a challenge.

Mesoporous trimetallic nanostructures, as an emerging class of multifunctional materials, have attracted great interest due to their unique structural properties. These mesoporous nanoarchitectures combine the properties of different metal elements to form a unique electronic structure that not only enhances the reactivity,^10^ but also improves the stability^11,12^ and selectivity^13^ of the catalyst. The mesoporous structure provides a large number of accessible active sites,^14^ which, together with the high internal surface area,^15^ provides sufficient contact opportunities for the reactant molecules. The cases involving Au-, Pd-, and Pt-based core–shell mesoporous structures are particularly intriguing due to their remarkable optical and catalytic activities. So far, various important trimetallic nanostructures containing Pd, Pt, and/or Au have been successfully prepared with controlled size and shape. For example, a facile micelle-assisted method using Au nanowires as seeds can prepare Au@PdPt nanowires with a mesoporous PdPt alloy shell.^16^ Similarly, a template method employing Au nanorods as seeds can produce Au@PdPt nanorods with a mesoporous PdPt shell.^17^ Additionally, mesoporous PdPt@Pt nanorings with high loading and good dispersion on reduced graphene oxide can be synthesized *via* a straightforward one-pot wet-chemical method.^18^ Lastly, seed-mediated wet chemical approaches can yield mesoporous Au nanotriangles with AgPt alloy mesoporous walls.^19^ Despite the successful demonstrations mentioned above, the multiple steps, high reaction temperatures, and/or prolonged durations complicate the synthetic procedures, making them challenging to scale up. Furthermore, achieving precise positional control of Au in Au/Pd/Pt trimetallic mesoporous nanostructures remains a significant difficulty.

Combining plasmonic Au with catalytically active metals like Pt and/or Pd within the same nanoparticle represents a versatile approach for constructing high-performance catalysts.^20^ In addition, Au-containing mesoporous structures also hold great promise for various other photodynamic and plasmonic photothermal applications.^21,22^ The localized surface plasmon resonance (LSPR) of Au-based nanostructures can be adjusted by modifying their sizes, compositions, and geometric morphologies.^23–25^ However, the previous mesoporous structures prepared *via* seed growth method^16,17^ or one-pot reduction methods^26,27^ often feature Au as the core and PtPd as the shell, which to some extent limits the utilization efficiency of Au and complicates the adjustment of the spatial distance between Au entities. In trimetallic systems, Au is often the first to be reduced to form the core due to its high standard redox potential, while the other metals in the shell may inhibit the light-absorbing properties of Au and reduce its activity and efficiency in catalytic reactions.^26,27^ As a result, achieving precise positional control of Au in Au/Pd/Pt trimetallic mesoporous structures remains difficult. In addition, a deep understanding of how to optimize photo-electrocatalytic efficacy by precisely controlling the position of Au is still necessary.

Herein, we present a simple yet efficient method for synthesizing Pd@Pt@Au mesoporous nanoparticles (MNPs), where well-dispersed isolated Au islands are located on the surface of Pd@Pt MNPs. Triblock copolymer (PEO_100_–PPO_65_–PEO_100_, Pluronic 127) serves as a soft-template in an aqueous solvent system, while l-ascorbic acid (AA) is utilized for the chemical reduction of the Pd, Pt, and Au precursors and to facilitate the nucleation of the metals around the F127 micelle templates. The catalytic performances of the prepared catalysts in the methanol oxidation reaction (MOR) are also evaluated under both visible light irradiation and in the absence of light. MOR is a key reaction in direct methanol fuel cells (DMFCs), where the chemical energy of methanol is converted to electrical energy through methanol electro-oxidation. Therefore, MOR is selected as a model reaction in this study. The catalytic performance of Pd@Pt@Au MNPs for MOR surpasses that of Pd@Pt NPs and commercial Pt black, regardless of light irradiation. Furthermore, under light irradiation, Pd@Pt@Au MNPs exhibit even higher MOR catalytic activity compared to the reference Au@Pd@Pt MNPs (Fig. S1†), where the core is composed of Au, the middle shell is Pd, and the outer shell is Pt. This indicates that well-dispersed isolated Au islands on the outer shell efficiently enhance surface plasmon resonance. This study presents a synthetic approach for precisely controlling the distribution and positioning of Au in trimetallic systems. It offers insights into the structure–performance relationship of Au-based nanostructures in plasmonic photothermal applications, providing valuable guidance for deeper understanding in this field. Our findings offer valuable insights into material design strategies that could inform the development of future systems where light-enhanced catalysis may be more viable, potentially leading to innovative solutions for practical applications, such as DMFCs.

## Results and discussion

**Fig. 1:**
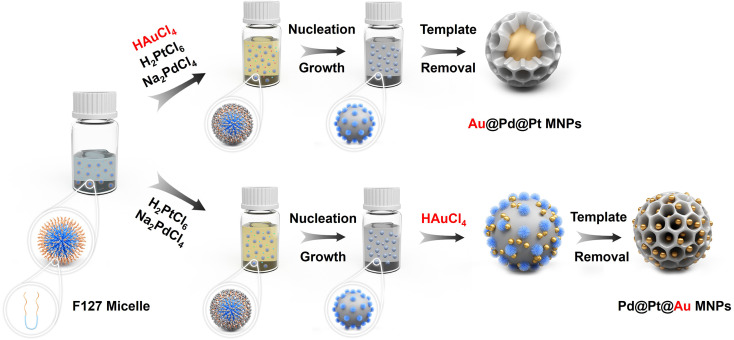
Illustration of different formation mechanism pathways of core–shell Au@Pd@Pt (up) and Pd@Pt@Au (down) mesoporous nanoparticles (MNPs). The synthetic process of Pd@Pt@Au MNPs can be divided into following steps: (1) addition of aqueous metal solutions causes the F127 copolymers to self-assembly into spherical micelles. The metal ions form aqua-complexes with the EO moieties of the PEO group *via* hydrogen bonding, *etc*. (2) The [PdCl_4_]^2−^ and [PtCl_6_]^2−^ ions are co-reduced, initiating nucleation. The particles then grow and gradually envelop the micelle templates. (3) The [AuCl_4_]^−^ species are introduced into the reaction solvent, where they adhere to the surface of deposited Pd@Pt, subsequently nucleating into Au islands. (4) The templates are finally removed by simple solvent extraction.

The flowchart in Fig. 1 outlines the synthesis procedure for the typical Pd@Pt@Au MNPs and reference Au@Pd@Pt MNPs. Upon addition of aqueous hydrochloric acid solution and metal precursors, pore directing F127 agent condensed to form spherical micelles with hydrophobic poly (propylene oxide) (PPO) cores surrounded by hydrophilic poly (ethylene oxide) (PEO) shells. Both metal precursors (H_2_PtCl_6_ and Na_2_PdCl_4_) and the AA were combined with the micelle solution, in which the PEO can bind to the dissolved metal species through hydrogen bonding, *etc*., incorporating them into the PEO shell. Subsequently, the dissolved [PdCl_4_]^2−^ and [PtCl_6_]^2−^ ions were reduced with AA at 40 °C, resulting in nucleation and growth of Pd@Pt on the micelle surfaces. Then, Au precursors were added and reduced without removing the F127 micelles. Finally, the resulting Pd@Pt@Au MNPs sample was obtained by removing the micelles. The reference Au@Pd@Pt MNPs were prepared by co-reducing all the metal precursors, including aqueous solutions of HAuCl_4_, H_2_PtCl_6_, and Na_2_PdCl_4_. Please see the Experimental section for details.

**Fig. 2:**
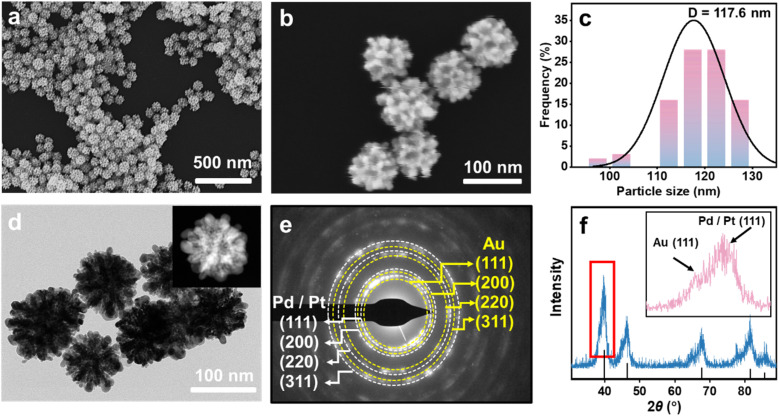
Characterization of Pd@Pt@Au MNPs. (a) Low and (b) high-magnification SEM image, (c) the corresponding particle size distribution, (d) TEM image and (inset) HAADF-STEM image, (e) SAED patterns obtained from the single Pd@Pt@Au MNP, and (f) wide-angle XRD pattern of Pd@Pt@Au MNPs and (inset) the enlarged XRD pattern of the selected area.

Fig. 2a and b display low- and high-magnification scanning electron microscopy (SEM) images of Pd@Pt@Au MNPs. In contrast to the Au@Pd@Pt MNPs which feature well-defined mesopores uniformly distributed over the entire external surface of the particles (Fig. S1†), the Pd@Pt@Au MNPs exhibit a similar narrow size distribution but are partially covered with isolated Au islands. The average particle diameter of Pd@Pt@Au MNPs is approximately 117.6 nm (Fig. 2c), which is relatively larger than that of Au@Pd@Pt MNPs, around 111.8 nm (Fig. S1†). The formation of these isolated Au islands is facilitated by providing sufficient nucleation sites for the reduction of Au ions on the surface of Pd@Pt. The gradual reduction of Au species results in the continuous nucleation and growth of isolated Au islands on the Pt outer layer.

**Fig. 3:**
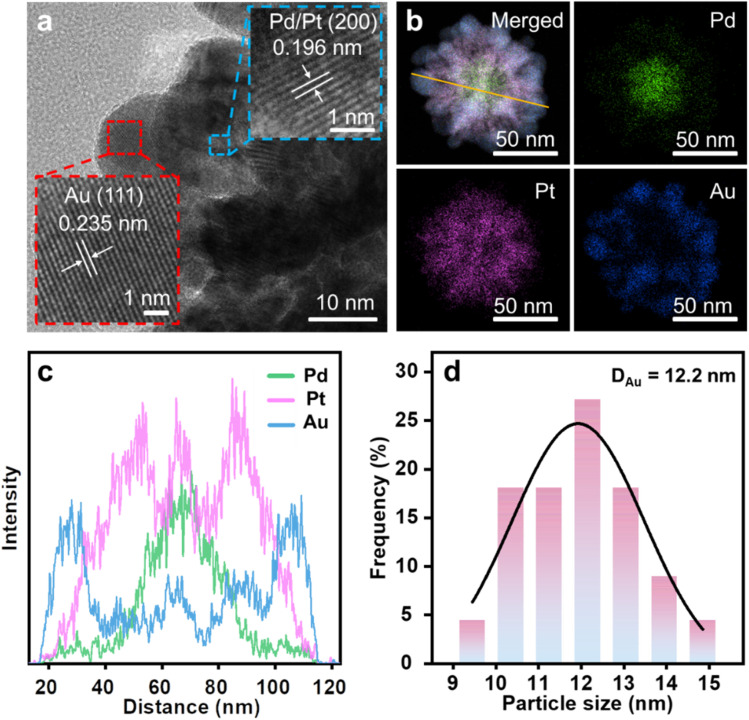
(a) TEM image, (b) HAADF-STEM image and corresponding elemental mappings, (c) line-scanning compositional profile, and (d) size distributions of Au islands in Pd@Pt@Au MNPs. The two insets in panel (a) are the enlarged area by red and blue dashed square panels.

For a more detailed examination of the distribution of Pd, Pt, and Au atoms, the Pd@Pt@Au MNPs were analyzed by using transmission electron microscopy (TEM) (Fig. 2d). The selected-area electron diffraction (SAED) patterns reveal two types of concentric rings of spots, which are indexed to the typical metallic face-centered cubic (*fcc*) structure of both PdPt and *fcc* Au, indicating a polycrystalline structure (Fig. 2e). The white concentric rings represent the PdPt, reflecting the very low lattice mismatch between Pt and Pd, which makes them difficult to distinguish. As shown in Fig. 3a, the distinct lattice fringes indicate a high crystallinity of the Pd@Pt@Au MNPs. The measured lattice distances of 0.196 nm and 0.235 nm are respectively attributed to the *fcc* (200) plane of PtPd and the *fcc* (111) plane of Au.

Wide-angle XRD measurements were employed to assess the crystal structures of the Pd@Pt@Au MNPs. The results revealed randomly oriented *fcc* crystals, with four peaks assigned to the (111), (200), (220), and (311) facets of crystal diffraction. The inset image in Fig. 2f displays two characteristic (111) diffraction peaks corresponding to the *fcc* crystal system of both Au and the PdPt. This observation also confirms the presence of the PdPt and Au as two distinct phases in the aforementioned selected area electron diffraction (SAED) patterns (Fig. 2e). Due to the lattice mismatch factor (0.77% for Pt–Pd *versus* 4.08% for Pt–Au), resolving the peaks of Pt and Pd in the XRD pattern is challenging, whereas the peaks of Au and Pt–Pd can be readily distinguished. For comparison, the XRD pattern of Au@Pd@Pt MNPs, where the core is composed of Au, the middle shell is Pd, and the outer shell is Pt, exhibits the same diffraction peaks; however, the diffraction peak of Au is more pronounced, indicating the formation of larger-sized crystals (Fig. S2†).

Elemental mapping images were collected using high-angle annular dark-field scanning TEM (HAADF-STEM) combined with energy-dispersive X-ray spectroscopy (EDS) for single Pd@Pt@Au MNPs (Fig. 3b and S3†). EDS mapping images illustrate the distribution of Pd, Pt, and Au in the particles, with Au being well-deposited on the outside of Pd@Pt MNPs. It is confirmed that this metallic distribution shows a clear delineation of the three metallic layers. The inner core consists mainly of Pd, while Pt occupies the medium layer, suggesting that the Pd precursor is preferentially reduced compared to the Pt precursor. This observation cannot be explained solely by the standard reduction potentials of Pt and Pd metal precursors (Table S1†). Such a Pd preferential deposition (*i.e.*, anomalous deposition) has occurred, contrary to the standard reduction potential order.^26,27^ This is likely due to complex deposition conditions, including the influence of additives and mixed metal precursors. Moreover, the line-scanning profile of the Pd@Pt@Au MNPs provides additional insight into their elemental composition, consistent with the elemental mapping results (Fig. 3c). The average diameter of isolated Au islands is approximately 12.2 nm, as calculated from HAADF-STEM images of multiple Pd@Pt@Au MNPs (Fig. 3d and S4†). To further validate the mesostructural orderings, small-angle X-ray scattering (SAXS) was employed (Fig. S5†). The peak of Pd@Pt@Au MNPs is also observed at *q* = 0.3095 nm^−1^, corresponding to a pore-to-pore distance of 20.30 nm.

TEM images along with EDS mapping of single and multiple Au@Pd@Pt MNPs corresponding to the STEM images reveal distinct distributions of Pd, Pt, and Au (Fig. S6–S9†). The Au core is observed as the inner core, while Pd and Pt constitute the outer layer. Elemental mapping images and line-scanning compositional profiles of Au@Pd@Pt MNPs further support this unique Au-core, Pd-inner layer, and Pt-outer layer structure. A distinct SAXS peak of Au@Pd@Pt MNPs was observed at *q* = 0.3005 nm^−1^ (Fig. S5†), corresponding to a pore-to-pore distance of 20.91 nm, which is well consistent with the pore-to-pore distance in the SEM results (Fig. S1†).

The differing nanostructures of Pd@Pt@Au MNPs and Au@Pd@Pt MNPs contribute to variations in particle diameters. The Pd@Pt@Au MNPs with isolated Au islands exhibit relatively larger diameters due to the additional diameter contributed by the Au islands (Fig. S10†). The mass ratios of Pt/Pd/Au are 67.6 : 7.5 : 24.9 for Au@Pd@Pt MNPs and 67.4 : 7.1 : 25.5 for Pd@Pt@Au MNPs, respectively, as confirmed by inductively coupled plasma-optical emission spectrometry (ICP-OES) (Table S2 and Fig. S11†). This result indicates successful and precise fabrication of trimetallic Pd@Pt@Au MNPs and Au@Pd@Pt MNPs, with nearly identical concentrations of different metallic portions.

**Fig. 4:**
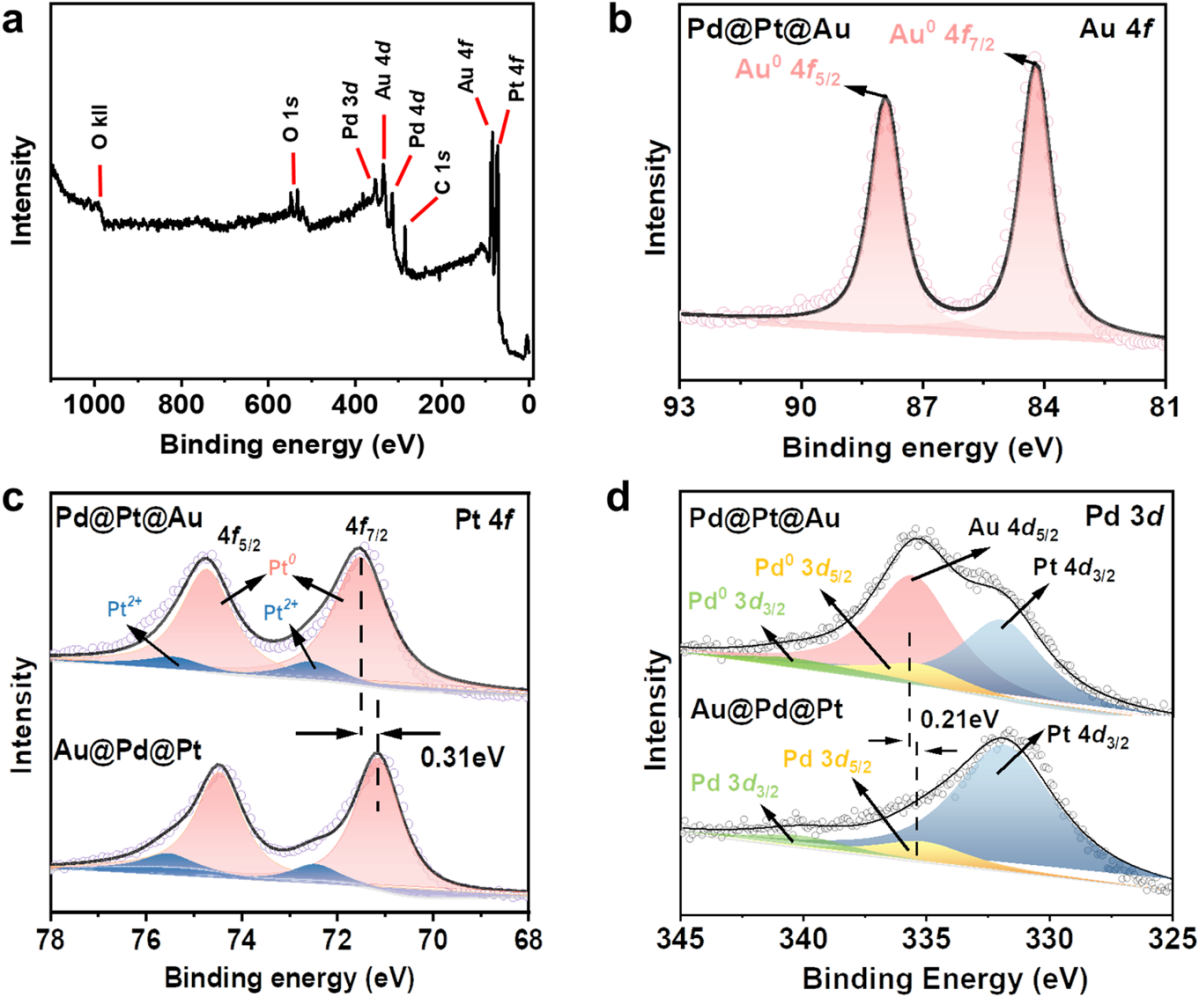
(a) Full scan spectrum and (b) Au 4f spectra of Pd@Pt@Au MNPs. Comparison of the (c) Pt 4f and (d) Pd 3d of Pd@Pt@Au and Au@Pd@Pt MNPs.

X-ray photoelectron spectroscopy (XPS) was employed as a surface-sensitive probe to investigate the chemical states and compositions of the Pd@Pt@Au MNPs and Au@Pd@Pt MNPs surfaces. The XPS survey spectrum of the Pd@Pt@Au MNPs confirms the presence of the four main composing elements, namely Pt, Pd, and Au (Fig. 4a). The Pt 4f high-resolution spectra of Pd@Pt@Au MNPS exhibited two peaks corresponding to the 4f_7/2_ and 4f_5/2_ doublet at 71.5 and 74.6 eV, respectively, consistent with metallic Pt (Fig. 4c). Additionally, two weak peaks located at 72.4 and 75.5 eV indicate partial oxidation of surface Pt. The Pt 4f binding energy of the Au@Pd@Pt MNPs sample is shifted in the positive direction compared with that of Pd@Pt@Au MNPs by 0.31 eV, suggesting more electronic interactions between the Pt middle layer and Au outer islands. The similar result is also observed in Pd 3d spectrum (Fig. 4d). The Pd 3d spectrum of Pd@Pt@Au MNPs revealed two peaks corresponding to the 3d_3/2_ and 3d_5/2_ doublet at 335.2 and 340.9 eV, respectively, in good agreement with literature values (335.5 and 341.1 eV) of metallic Pd.^28^ The Pd 3d high-resolution spectra of Au@Pd@Pt MNPs exhibited two peaks corresponding to the 3d_5/2_ and 3d_3/2_ doublet at 335.4 and 341 eV, respectively, also consistent with zerovalent Pd. Besides, the binding energy of Pd^0^ in Pd@Pt@Au MNPs also exhibits a negative shift of 0.21 eV relative to Au@Pd@Pt MNPs, indicating that Pd acts as an electron donator due to interfacial interaction with Au. Due to the relatively low amount and inner position of Pd in the trimetallic system, the Pd peaks are not clearly observed in both samples, while Au 4d_5/2_ peak is observed in Pd@Pt@Au MNPs. In Au 4f high-resolution spectra, two peaks of Pd@Pt@Au MNPs corresponding to the 4f_7/2_ and 4f_5/2_ doublet at 84.2 and 87.8 eV, respectively, are assigned to zerovalent Au (Fig. 4b). No peaks are observed in the high-resolution spectra of Au@Pd@Pt MNPs, as the Au core is covered by both Pd and Pt shells (Fig. S12†). These data exhibit the different geometrical positions of these three metals in NPs. The molar ratios of Pt, Pd, and Au in Pd@Pt@Au and Au@Pd@Pt MNPs are critical for understanding their catalytic performance and structural characteristics. However, since XPS only provides surface composition information, it is not a reliable method for assessing the overall compositional ratios in core–shell MNPs. Therefore, in this study, we employed ICP-OES analysis to accurately quantify the relative amounts of Pt, Pd, and Au in the samples (Fig. S11 and Table S2†).

**Fig. 5:**
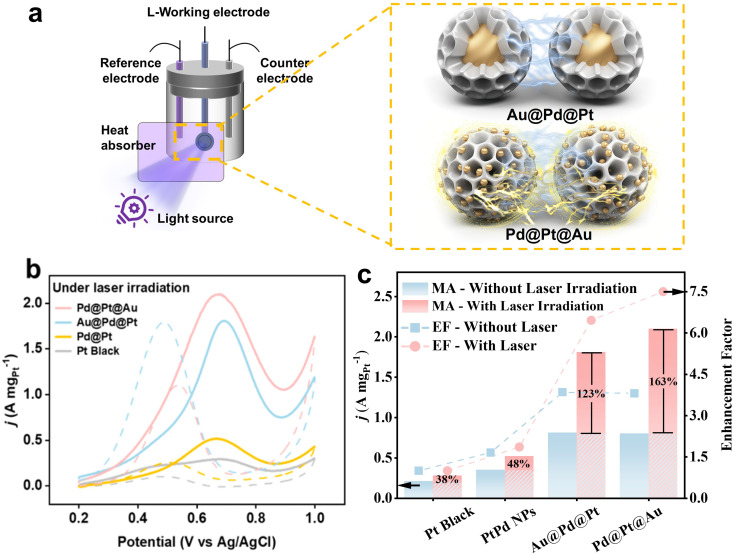
(a) Brief illustration of experimental set-up and different light-induced plasmon-driven interaction of MNPs with different Au localization, (b) geometrical area-normalized CV curves for MOR catalyzed by Pd@Pt@Au MNPs, Au@Pd@Pt MNPs, Pd@Pt MNPs, and PtB respectively in 0.5 M H_2_SO_4_ containing 0.5 M methanol. All the CV curves were obtained at a scan rate of 50 mV s^−1^. The light source with deep UV and visible light (300 to 630 nm) is applied. (c) Enhancement ratio of four catalysts before and after photoinduction.

The electrocatalytic performance of Pd@Pt@Au MNPs towards the MOR was evaluated in comparison to Au@Pd@Pt MNPs, Pd@Pt MNPs (without Au loading), and commercial Pt black. Two experimental conditions were applied: one with ultraviolet-visible (UV-vis) light irradiation and the other without. In contrast to traditional MOR tests, a light source, MAX-303 Xenon Light Source 300 W, equipped with various filters ranging from 300 to 630 nm (deep UV light and visible light) and 450 to 630 nm (visible light only), was introduced (Fig. 5a). A heat absorber was employed to mitigate the influence of light-induced heat on the experiment.

The MOR activities were compared by normalizing peak currents in geometric area of working electrode (Fig. S13†) and Pt mass loading for all samples (Fig. 5b and S14†). Mass-normalized cyclic voltammetry (CV) curves for MOR were measured in 0.5 M H_2_SO_4_ solution containing 0.5 M CH_3_OH at a sweep rate of 50 mV s^−1^. The peak current density is conventionally employed to assess catalytic activity in MOR. The results clearly demonstrate that the current densities of Pd@Pt@Au and Au@Pd@Pt MPNs are superior to those of Pd@Pt MNPs and commercial Pt black without light induction. The compositions and current density values in the forward sweep are as follows: Pd@Pt@Au MNPs (0.80 A mg_Pt_^−1^), Au@Pd@Pt MNPs (0.81 A mg_Pt_^−1^), Pd@Pt NPs (0.35 A mg_Pt_^−1^), and PtB (0.21 A mg_Pt_^−1^) (Fig. 5c and S14†). The Pd@Pt@Au MNPs exhibit the highest catalytic activity, approximately 3.81 times higher than that of Pt black without light induction. When the light source with deep UV light and visible light (300 to 630 nm) is applied, the current density of Pd@Pt@Au MNPs (2.10 A mg_Pt_^−1^) in the forward sweep is significantly higher than those of Au@Pd@Pt MNPs (1.81 A mg_Pt_^−1^), Pd@Pt NPs (0.52 A mg_Pt_^−1^), and PtB (0.28 A mg_Pt_^−1^) (Fig. 5c and S14†). Pd@Pt@Au MNPs demonstrate the greatest catalytic activity, approximately 7.50 times higher than that of PtB, while Au@Pd@Pt MNPs show an increase of 6.46 times. Even after normalizing the current density in the forward sweep to the geometric area of the glassy carbon electrode (GCE) where an equal amount of catalyst is applied, the current density of Pd@Pt@Au MNPs (10 mAcm_geo_^−2^) is higher than those of Au@Pd@Pt MNPs (8.50 mA cm_geo_^−2^), Pd@Pt NPs (2.75 mA cm_geo_^−2^), PtB (2.05 mA cm_geo_^−2^) with light irradiation (Fig. S13†). The current density of Pd@Pt@Au MNPs is around 4.87 times and 2.66 times higher than that of PtB, respectively, with or without light irradiation when normalized by the geometric area of the working electrode. When the light source with visible light only (450 to 630 nm) is applied, the values of Au@Pd@Pt MNPs and Pd@Pt NPs are two-thirds of those with deep UV light and visible light (300 to 630 nm). However, regardless of light source, the values of Pd@Pt MNPs and PB NPs show similar values.

During visible light excitation with deep UV light and visible light, the mass activity of all samples is greatly increased, with the Pd@Pt@Au MNPs exhibiting the highest increase at 163%, as shown in Fig. 5c. In comparison, the MA of Au@Pd@Pt MNPs, Pt@Pt NPs, and PtB increased by 123%, 48%, and 38%, respectively (Fig. 5c). However, when calculating the EF without light irradiation, the values for Au@Pd@Pt and Pd@Pt@Au MNPs are almost the same. This result suggests that the presence of Au islands on the outer shell leads to a significantly higher EF of MOR during light irradiation compared to Au@Pd@Pt MNPs with Au cores.

Both Pd@Pt@Au MNPs and Au@Pd@Pt MNPs exhibit superior catalytic performance compared to the electrocatalysts containing only Pd and Pt (*i.e.*, Pd@Pt MNPs and PtB), regardless of light induction. The integration of plasmonic metals like Au with catalytically active metals such as Pt and Pd within a single nanoparticle offers a versatile strategy for developing high-performance catalysts. For example, incorporating Au with Pd/Pt enhances the resistance to CO poisoning. As shown in Fig. S13 and S14,† Pd@Pt@Au MNPs show a higher *I*_f_/*I*_b_ ratio compared to other samples, indicating greater tolerance to poisoning by ethanol intermediates. Poisoning is a significant challenge in the MOR, as CO strongly adsorbs onto the catalyst surface, blocking active sites and inhibiting MOR. This greatly reduces both the catalytic efficiency and the durability of the catalyst. The binding energy of molecular CO and other adsorbates tends to be weaker on Au/Pt surfaces compared to Pt alone.^29–31^ As shown in Fig. S15,† the UV-vis absorption spectra of Pd@Pt@Au MNPs and Au@Pd@Pt MNPs exhibit distinct shapes, highlighting the difference in the positioning of Au within these two systems. The Pd@Pt@Au MNPs exhibit a distinct absorbance peak at approximately 551 nm, consistent with prior research on Au colloid seeds.^32^ The Au@Pd@Pt MNPs exhibit no distinct absorbance peak at approximately 551 nm when compared to Pd@Pt@Au MNPs. However, their overall absorbance in this region is higher than that of Pd@Pt MNPs. All three MNPs exhibit a clear absorbance peak at approximately 300 nm. Under light irradiation, the Pd@Pt@Au MNPs and Au@Pd@Pt MNPs demonstrate enhanced photocatalytic MOR compared to conditions without plasmonic excitation. The plasmonic effect of Au is expected to induce both photothermal and photo-electronic effects. We believe that the enhanced current density can be attributed to the photothermal and/or photo-electronic effects, induced by hot electron–hole pairs generated through localized surface plasmon resonance (LSPR) excitation.^33^ Because the temperature of the methanol aqueous system is maintained at room temperature due to the presence of a heat absorber, the observed enhancement in current density is primarily attributed to the photothermal and/or photo-electronic effects, induced by LSPR in our system. It is worth noting that the sensitivity of LSPR can vary depending on the size of the NPs.^34^ Two distinct regimes can be identified in Fig. 5a. Both LSPR and SPR sensitivities can be enhanced by increasing the coverage of Au NPs, thereby reducing interparticle distances.^35^ The average size of the Au core in Au@Pd@Pt MNPs is approximately 30 nm (Fig. S6†), with interparticle distances (>100 nm, over the particle size of Au@Pd@Pt MNPs) (Fig. S1†). In contrast, the average size of the Au-islands deposited on the outer shell of Pd@Pt@Au MNPs is approximately 12.2 nm (Fig. 3d), with an interspace between the islands of a few nanometers (Fig. S4†). The distribution of Au-islands on the outer shell appears random and evenly spread. The close proximity between individual Pd@Pt@Au MNPs is carefully regulated to be below 5 nm, facilitating significant enhancement in plasmonic coupling and resonance for each Au island.^36,37^ The weaker absorbance peak around 551 nm for Au@Pd@Pt MNPs suggests that LSPR-induced effects may be less prominent in this system compared to Pd@Pt@Au MNPs. This suggests that the presence of Au within the core contributes to light absorption, but its plasmonic resonance is weakened due to its encapsulation within the Pd@Pt shell.

Further investigation into the exceptional performance of Pd@Pt@Au MNPs in MOR under light irradiation was conducted. As shown in Fig. S15,† all three MNPs exhibit a clear absorbance peak at approximately 300 nm. As shown in Fig. S13c and d,† the peak currents of Pd@Pt MNPs and PB NPs increase slightly under light irradiation, including deep UV wavelengths, compared with conditions without deep UV light. In contrast, the peak currents of Pd@Pt@Au MNPs and Au@Pt@Pd MNPs increase by up to 50% under light irradiation, including deep UV wavelengths, compared with no deep UV. Although the local temperature on the catalyst surface can directly increase significantly due to the thermal effect of light heating under UV light irradiation, in this study this effect is minor compared to the contribution of LSPR. The above observations suggest that the exceptional performance of Pd@Pt@Au MNPs in MOR under light irradiation is predominantly driven by plasmonic effects rather than direct thermal effects.

**Fig. 6:**
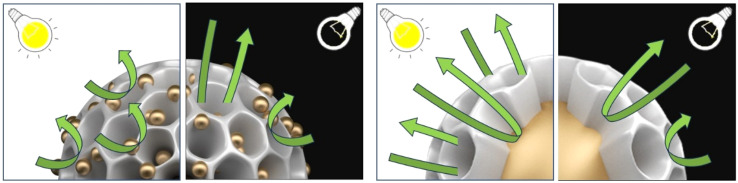
Illustration of the MOR mechanism at Pd@Pt@Au and Au@Pd@Pt, both with and without light irradiation.

To further analyze the effect of Au NPs (*e.g.*, particle size, distance, and position) within the Pd@Pt@Au and Au@Pd@Pt MNPs on LSPR and SPR, electrochemical impedance spectroscopy (EIS) was employed to evaluate their catalytic activity for MOR under both light irradiation and non-irradiated conditions (Fig. S16†). The interpretation of Nyquist plots in Fig. S16† is summarized in the note section. This significant change in impedance under light irradiation cannot be attributed solely to the thermal effect of light heating. Therefore, the enhanced current density can be explained by photothermal and/or photo-electronic effects, induced by LSPR. The difference in the LSPR excitation effect between the MNPs is likely due to variations in the Au particle size, distance, and position within the MNPs, as indicated by the EIS results. For Pd@Pt@Au MNPs, MOR is significantly accelerated by LSPR under light irradiation. The impedance responses at low frequency, which correspond to diffusion resistance, dramatically decrease. This suggests that MOR occurs preferentially at shell position, where uniformly isolated and dispersed Au islands contribute to enhanced reaction kinetics with minimal diffusion limitations, compared to Au-core position (Fig. 6). The well-distributed Au islands on the outer shell likely promote catalytic activity at the shell surface reduce mass transport resistance, leading to a greater enhancement in MOR. In contrast, for Au@Pd@Pt MNPs, MOR is also accelerated by LSPR under light irradiation. However, the impedance responses at low frequencies, which correspond to diffusion resistance, exhibit only slight changes. This suggests that MOR enhances near the Au core position, where diffusion limitations remain comparable to non-irradiated conditions (Fig. 6). The encapsulation of Au within the Pd@Pt shell may hinder efficient charge transfer and LSPR-driven diffusion enhancements, resulting in a less pronounced improvement in MOR compared to Pd@Pt@Au MNPs. Furthermore, by comparing these two results, it was not possible to distinguish between the contributions of the photothermal effect and the photo-electronic effect. However, when comparing Pd@Pt@Au and Au@Pd@Pt, a simple photothermal effect alone cannot account for the significant decrease in diffusion resistance relative to the increase in reaction rate when Au islands are located on the outer surface. This suggests that the relative contribution of the photo-electronic effect increases when Au particles are present on the outer surface.

Overall, our finding indicates that positioning Au, especially uniformly isolated and dispersed Au islands, on the outer shell results in superior performance in plasmon-induced photocatalysis across the entire system, even when compared to recently reported MOR electrocatalysts (Table S3†).

We also conducted stability tests using chronoamperometry (CA) at a constant potential of 0.65 V (*vs.* Ag/AgCl) to evaluate our catalysts' durability (Fig. S17†). The CA measurements show an initial rapid decline in mass activity, followed by stabilization at a plateau, primarily due to surface contamination and poisoning by intermediate species. Despite this initial drop, Pd@Pt@Au MNPs retain higher current density, outperforming Au@Pd@Pt MNPs and commercial PB, highlighting the superior MOR durability of Pd@Pt@Au MNPs.

## Conclusion

In this study, we have demonstrated a straightforward method for synthesizing Pd@Pt@Au MNPs with distinct core–shell structures, emphasizing precise control over the position and distribution of Au. Through a carefully designed synthesis procedure, the resulting Pd@Pt@Au MNPs show well-dispersed isolated Au-islands on their outer shell, contributing to an amplified plasmonic coupling and resonance effect. Our investigation reveals that the Pd@Pt@Au MNPs exhibit superior catalytic activity in the MOR compared to commercial PtB, displaying an approximate 7.4-fold enhancement in catalytic performance under light irradiation. The external positioning of Au on the shell of the Pd@Pt@Au MNPs offers superior plasmonic enhancement due to the small interspace distance of Au-islands and the well-spread but short distance between single Au islands, resulting in a stronger LSPR effect. This methodology provides valuable insights into the fabrication of trimetallic MNPs with balanced proportions of different metal components, addressing the challenge of random metal distribution encountered in previous approaches. Furthermore, it underscores the potential of precisely positioning plasmonic metals in spatial locations within mesoporous structures for enhanced LSPR effects in catalytic applications. Our findings offer valuable insights into metal-based material design strategies that will guide the development of future systems with enhanced light-driven catalysis, potentially leading to innovative solutions for various electrochemical applications such as DMFC and water splitting.^38–43^

## Experimental section

### Materials

All the chemicals and solvents used in the study were obtained from Sigma-Aldrich without any further purification. The materials included HAuCl_4_, H_2_PtCl_6_, Na_2_PdCl_4_, l-ascorbic acid (AA), methanol, and Pluronic 127 (PEO_100_–PPO_65_–PEO_100_). It is worth noting that in the Pluronic F127 molecule, PEO and PPO are poly(ethylene oxide) and poly(propylene oxide), respectively. To compare the electrochemical performance of the catalysts, commercial Pt black (PtB) was purchased from Alfa Aesar. Finally, the Nafion solution was obtained from Sigma-Aldrich.

### Preparation of Pd@Pt@Au mesoporous nanoparticles (MNPs)

To prepare the Pd@Pt@Au MNPs, 100 mg of F127 was dissolved completely in aqueous metal precursors, including H_2_PtCl_6_ (40 mM, 6 mL) and Na_2_PdCl_4_ (40 mM, 1.2 mL). Then, the following aqueous solutions were added in this sequence: hydrochloric acid (HCl) solution (0.1 M, 120 μL), and AA (100 mM, 6 mL) solution. The solution was mixed, and the vial was kept in a 40 °C-water bath for 4 hours, causing the solution to turn to black. After the water bathing, the aqueous HAuCl_4_ solution (40 mM, 2 mL) was added into the vial and was kept in a 40 °C-water bath for another 4 hours. In our synthesis, we selected ascorbic acid (AA) as the reducing agent due to its milder reducing power than sodium borohydride (NaBH_4_). The reaction conditions were optimized to achieve the desired Pd@Pt@Au MNP structure. The samples were collected by centrifugation at 14 000 rpm for 10 minutes and then resuspended in acetone and water. This washing/centrifugation cycle step was performed four times prior to characterization.

### Preparation of Pd@Pt MNPs

The preparation steps of Pd@Pt MNPs are the same previous work in our lab.^44^ 100 mg of F127 was dissolved completely in aqueous metal precursors, including H_2_PtCl_6_ (40 mM, 6 mL), and Na_2_PdCl_4_ (40 mM, 1.2 mL). Then, the following aqueous solutions were added in this sequence: hydrochloric acid (HCl) solution (0.1 M, 120 μL), and AA solution (100 mM, 6 mL). The solution was mixed, and the vial was kept in a 40 °C-water bath for 4 hours, causing the solution to turn to black. The samples were collected by centrifugation at 14 000 rpm for 10 minutes and then resuspended in acetone and water. This washing/centrifugation cycle step was performed four times prior to characterization.

### Preparation of Au@Pd@Pt MNPs

To prepare the Au@Pd@Pt MNPs as a reference,^44^ 100 mg of F127 was dissolved completely in aqueous metal precursors, including H_2_PtCl_6_ (40 mM, 6 mL), Na_2_PdCl_4_ (40 mM, 1.2 mL) and HAuCl_4_ (40 mM, 2 mL). Then, the following aqueous solutions were added in this sequence: hydrochloric acid (HCl) solution (0.1 M, 120 μL), and AA solution (100 mM, 6 mL). The solution was mixed, and the vial was kept in a 40 °C water bath for 4 hours, causing the solution to turn to black. The samples were collected by centrifugation at 14 000 rpm for 10 minutes and then resuspended in acetone and water. This washing/centrifugation cycle step was performed four times prior to characterization.

### Characterization

The samples were initially characterized by field-emission scanning electron microscopy (FESEM) and elemental mapping analysis using a Hitachi SU8000 at an accelerating voltage of 10 kV. Transmission electron microscopy (TEM), elemental mapping analysis, and high-angle annular dark-field scanning TEM (HAADF-STEM) were performed on a JEOL JEM-2100F with an energy-dispersive spectrometer. Wide-angle powder X-ray diffraction (XRD) patterns were recorded with a Rigaku Rint 2500 diffractometer using monochromated Cu-Kα radiation (40 kV, 30 mA) source. Small-angle X-ray scattering measurements (SAXS) were conducted with a Rigaku NANO-Viewer to evaluate the porous structures of the samples. XPS measurements were conducted using an Al Kα radiation source (JPS-9010), and the results were calibrated using the C 1s peak at 284.8 eV.

### Electrochemical measurement of methanol oxidation reaction (MOR)

Electrochemical measurements were conducted in a conventional three-electrode cell with Ag/AgCl (saturated KCl) as the reference electrode, a platinum wire as the counter electrode, and a modified glassy carbon electrode (GCE, 3 mm diameter) as the working electrode. CV curves were collected on a CHI 842B electrochemical analyzer (CHI Instruments, USA). To prepare the working electrode, the GCE was polished, coated with 5.0 μg of sample material (*e.g.*, Au@Pd@Pt MNPs, Pd@Pt@Au MNPs, Pd@Pt NPs, PtB), and dried at room temperature. The GCE was then covered with 5.0 μL of Nafion solution (0.05 wt%) and dried completely at room temperature. The GCE was electrochemically activated by cycling the potential between +0.2 and +1.0 V (*vs.* Ag/AgCl) in 0.5 M H_2_SO_4_. All MOR measurements were performed in a solution of 0.5 M H_2_SO_4_ containing 0.5 M methanol at a scan rate of 50 mV s^−1^. The geometric current density *j*_geo_ (mA cm_geo_^−2^) was normalized by the geometric area of the working electrode according to the following eqn (1):1
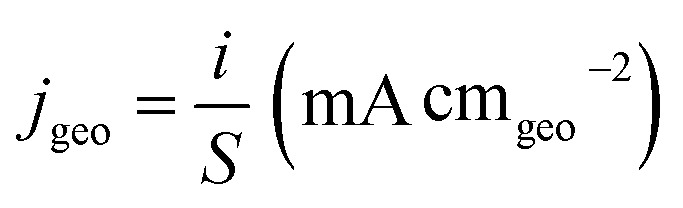
where *i* (mA) is the obtained current, and *S* is the geometric area of the working electrode (0.07065 cm^2^).

The mass activity of *j*_Pt_ (A mg^−1^) was obtained by normalizing the measured current with respect to the mass of Pt according to the following eqn (2):2
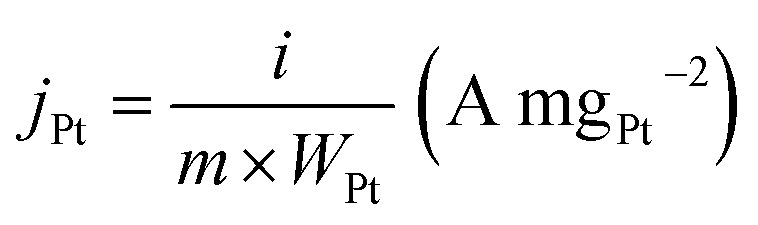
where *i* (A) is the obtained current, *W*_Pt_ is the weight percent of Pt (wt%) in the catalyst, and *m* is the loading of the catalyst on GCE.

### Electrochemical impedance spectroscopy for MOR

Electrochemical impedance spectroscopy (EIS) was performed using the same cell system, working electrode preparation, activation process, and electrolyte solution as described in the section on the electrochemical measurement of the MOR. EIS measurements were conducted using a SP-300 Potentiostat/Galvanostat system (Bio-Logic Science Instruments, France). Nyquist plots were obtained at the rest potential, the onset potential (defined as the potential at 0.1 mA mg_Pt_^−1^), and the peak current potential. EIS was carried out over a frequency range of 100 kHz to 10 mHz with an AC amplitude of 5 mV (zero-to-peak) at each set potential.

## Data availability

The data that support the findings of this study are available in the ESI.†

## Author contributions

L. Zhu and Y. Kang: conceptualization, methodology, conducting chemical experiments, and manuscript writing. Y. Zhao, D. Jiang and X. Wei: data curation and formal analysis. X. Xu and K. Nakagawa: assistance in collecting part of the experimental data. M. Eguchi, T. Yokoshima and Y. Yamauchi: supervision, manuscript review, and editing.

## Conflicts of interest

There are no conflicts to declare.

## Supplementary Material

SC-016-D4SC07345B-s001
