# Supplementary material for: Precise positioning of Au islands within mesoporous Pd–Pt nanoparticles for plasmon-enhanced methanol oxidation
Source: Chem Sci. 2025 Apr 9;16(19):8309–18. doi: 10.1039/d4sc07345b (PMC11979621; doi:10.1039/d4sc07345b)
Supplement: SC-016-D4SC07345B-s001 [file SC-016-D4SC07345B-s001.pdf]

Supplementary Information (SI) for Chemical Science.
This journal is © The Royal Society of Chemistry 2025

Supplementary Figures

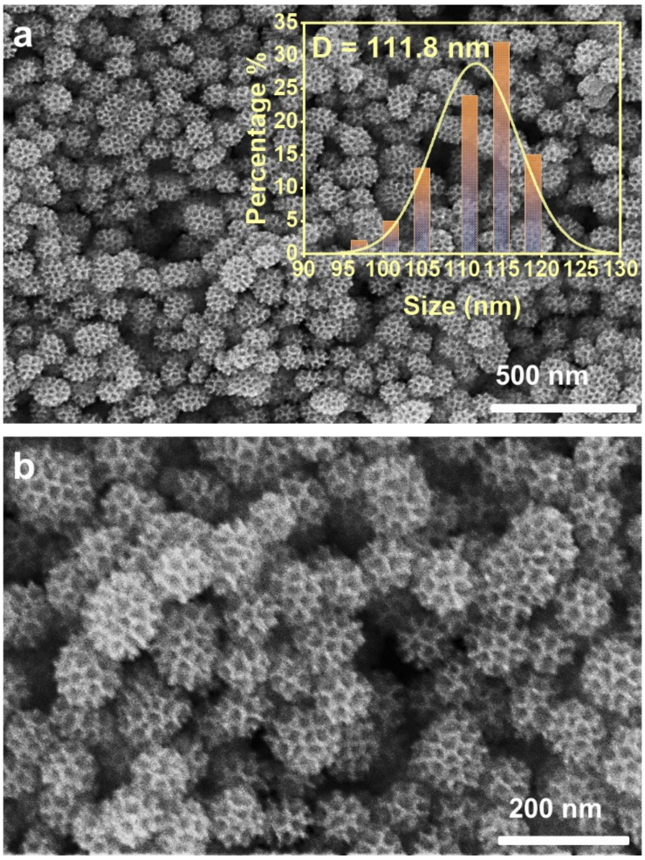

Figure S1. (a) Low- and (b) high-magnification SEM image of Au@Pd@Pt MNPs. The inset in panel (b) is the corresponding particles size distribution of Au@Pd@Pt MNPs.

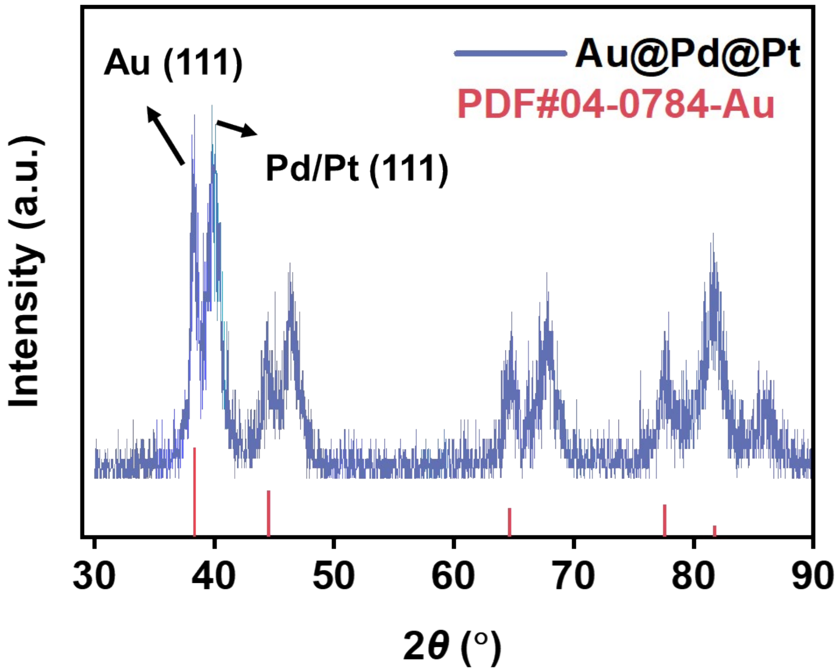

Figure S2. XRD patterns of Au@Pd@Pt MNPs.

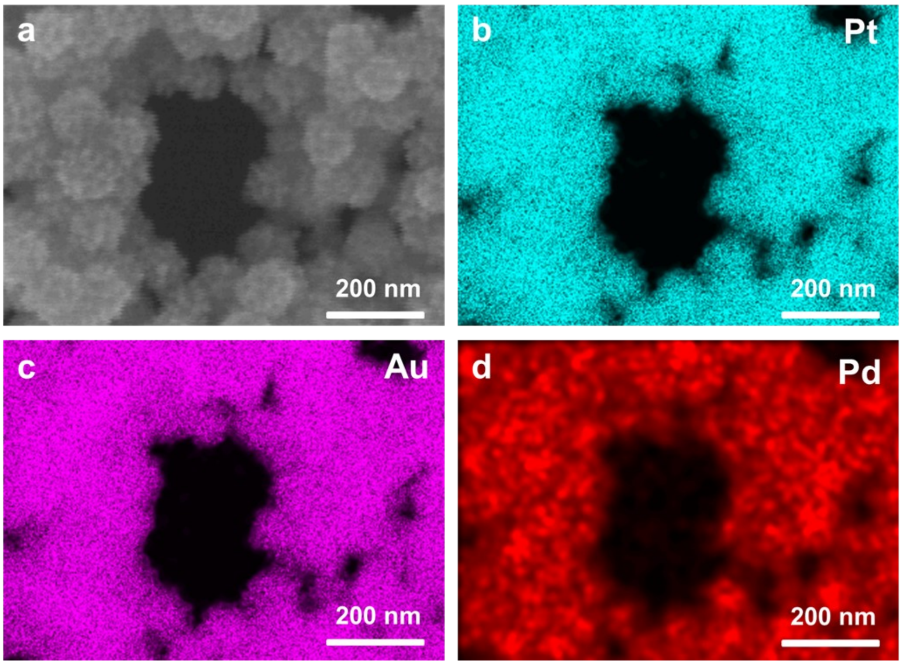

Figure S3. SEM image and corresponding SEM-EDS maps of Pd@Pt@Au MNPs.

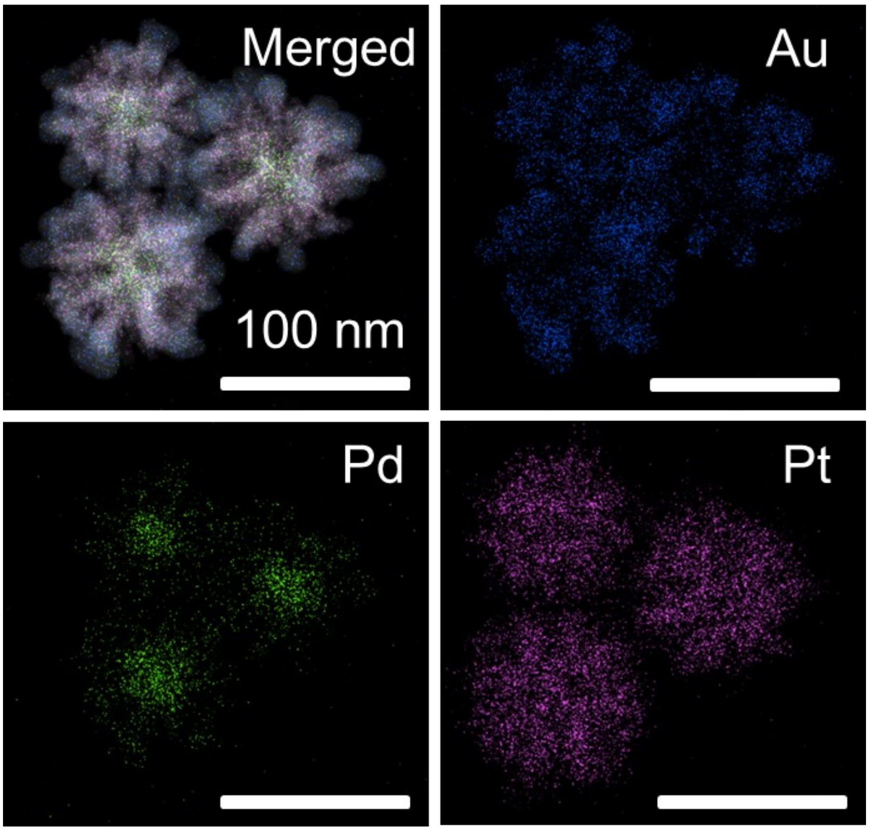

Figure S4. Low-magnification HAADF-STEM image and corresponding mapping of Pd@Pt@Au MNPs. Scale bars, 100 nm.

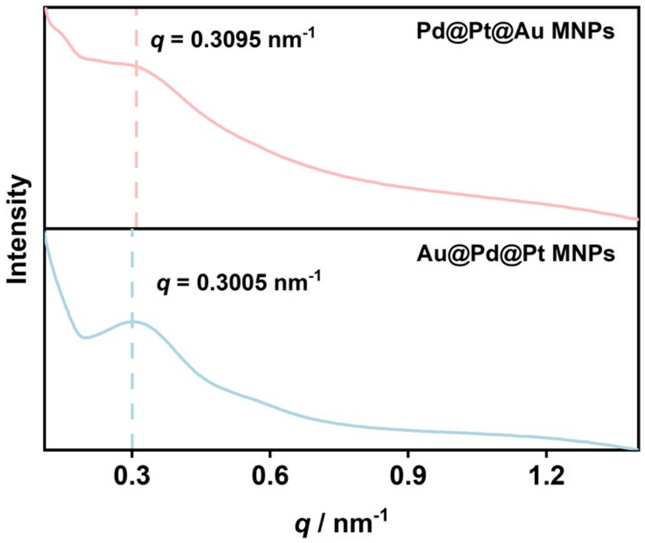

Figure S5. The SAXS pattern of Au@Pd@Pt MNPs and Pd@Pt@Au MNPs.

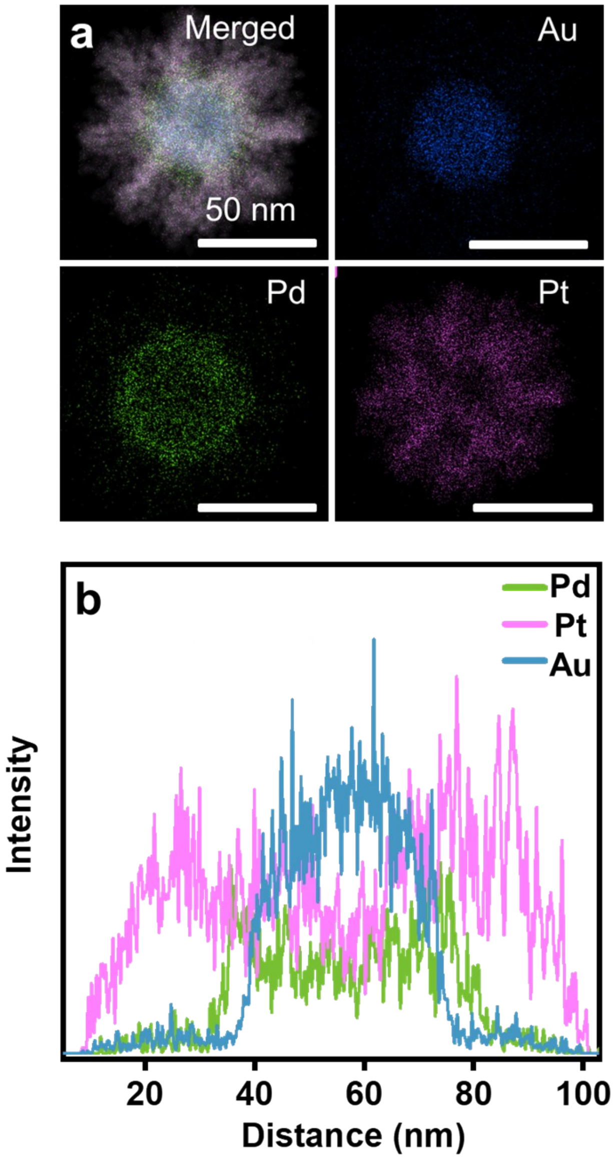

Figure S6. (a) High-magnification HAADF-STEM image and corresponding mapping of Au@Pd@Pt MNPs. Scale bars, 50 nm. (b) Line-scanning compositional profile of Au@Pd@Pt MNPs.

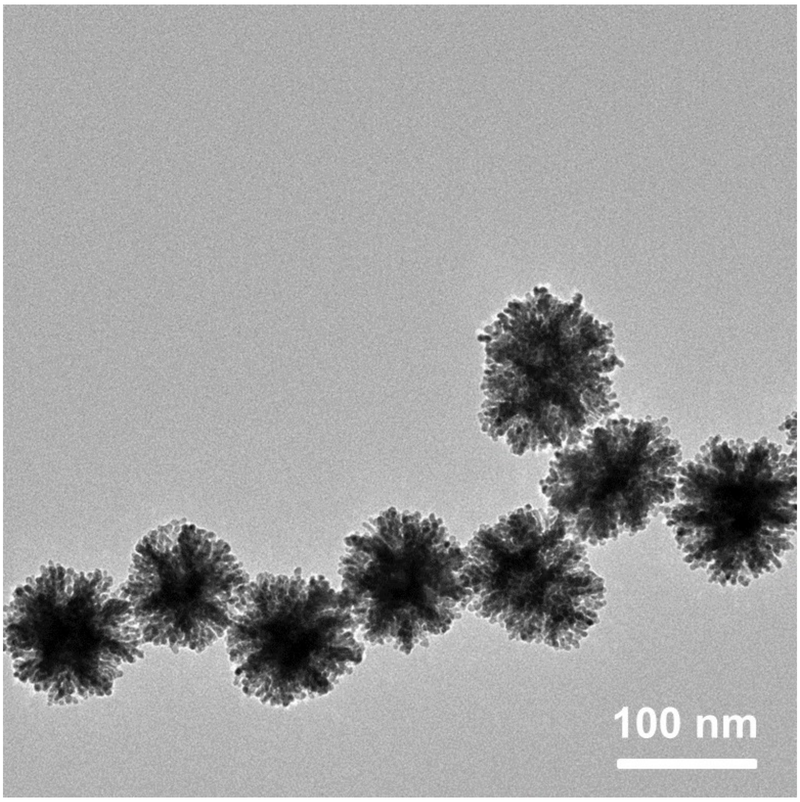

Figure S7. Low-magnification TEM image of Au@Pd@Pt MNPs.

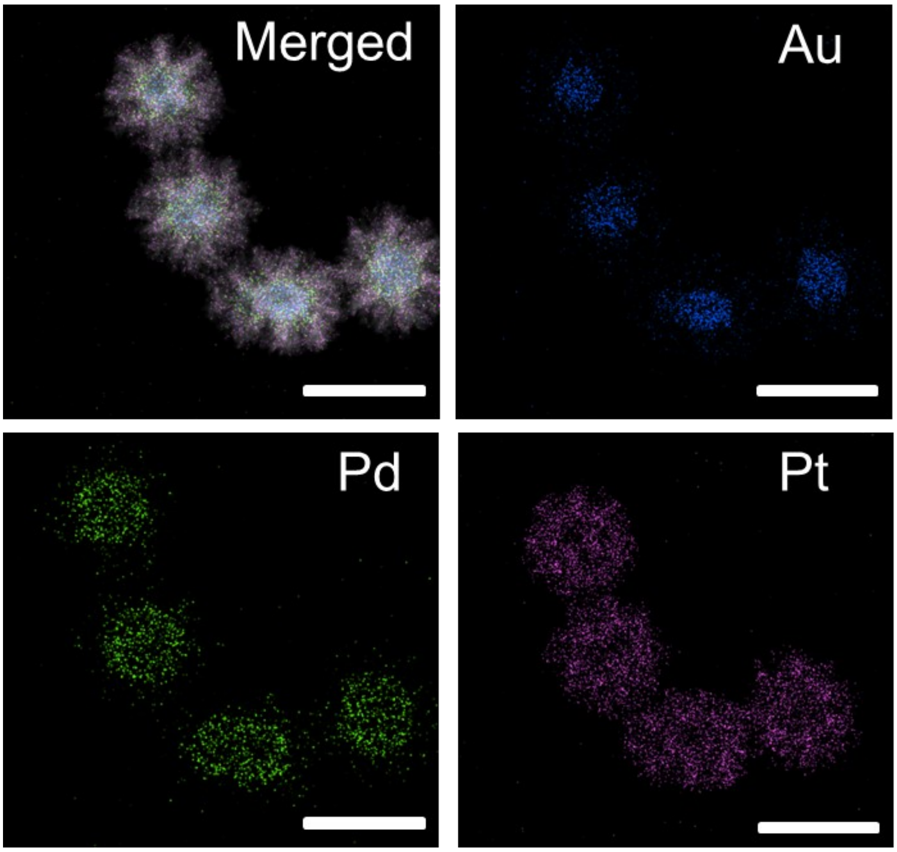

Figure S8. High-magnification HAADF-STEM image and corresponding mapping of Au@Pd@Pt MNPs. Scale bars, 100 nm.

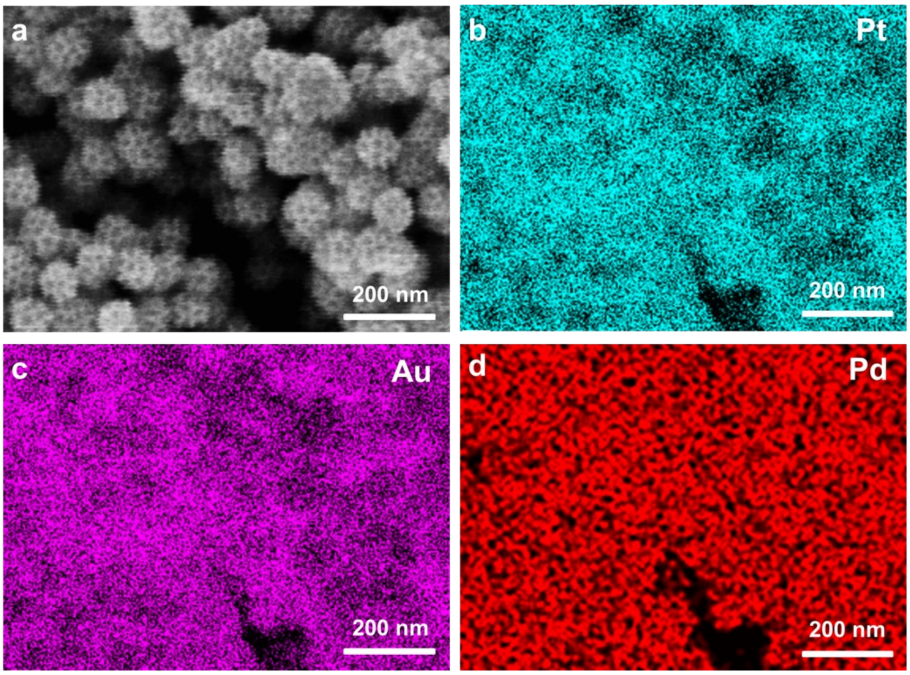

Figure S9. SEM image and corresponding SEM-EDS maps of Au@Pd@Pt MNPs.

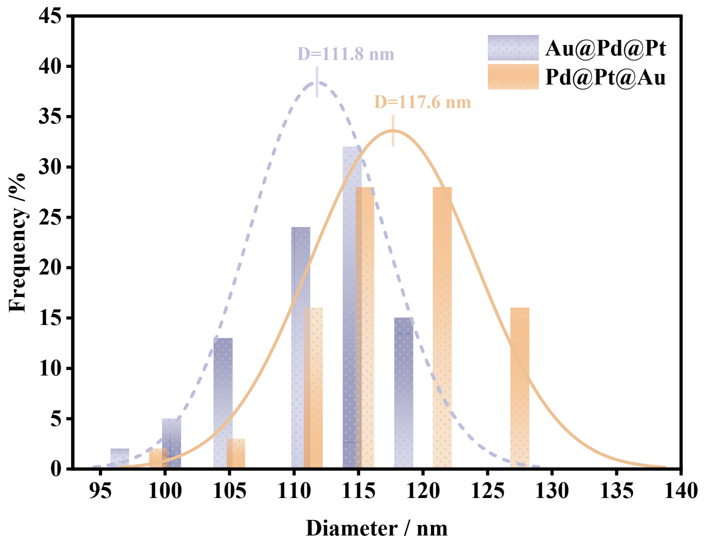

Figure S10. Particle size distributions of Pd@Pt@Au and Au@Pd@Pt MNPs.

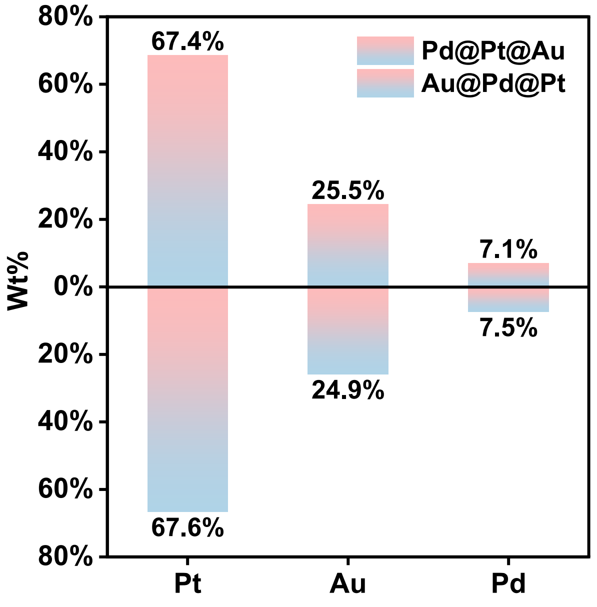

Figure S11. The comparison of ICP–OES analysis of Pd@Pt@Au MNPs and Au@Pd@Pt MNPs.

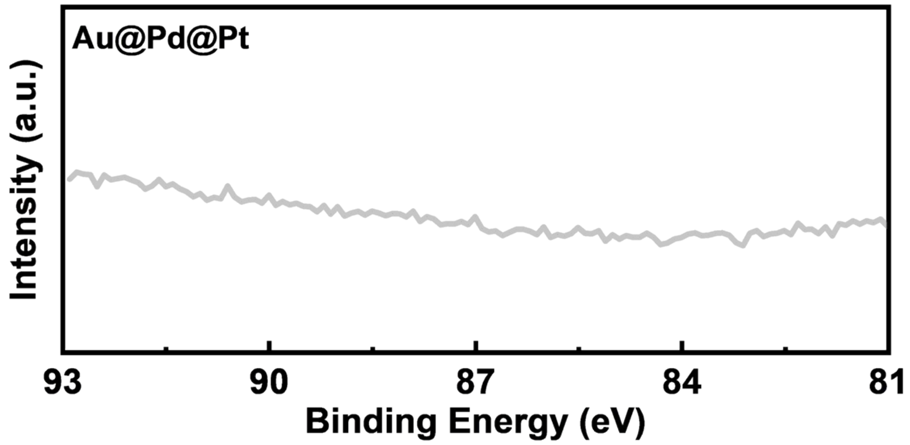

Figure S12. High resolution XPS spectra of Au in Au@Pd@Pt MNPs, showing a minimal amount of Au on the surface.

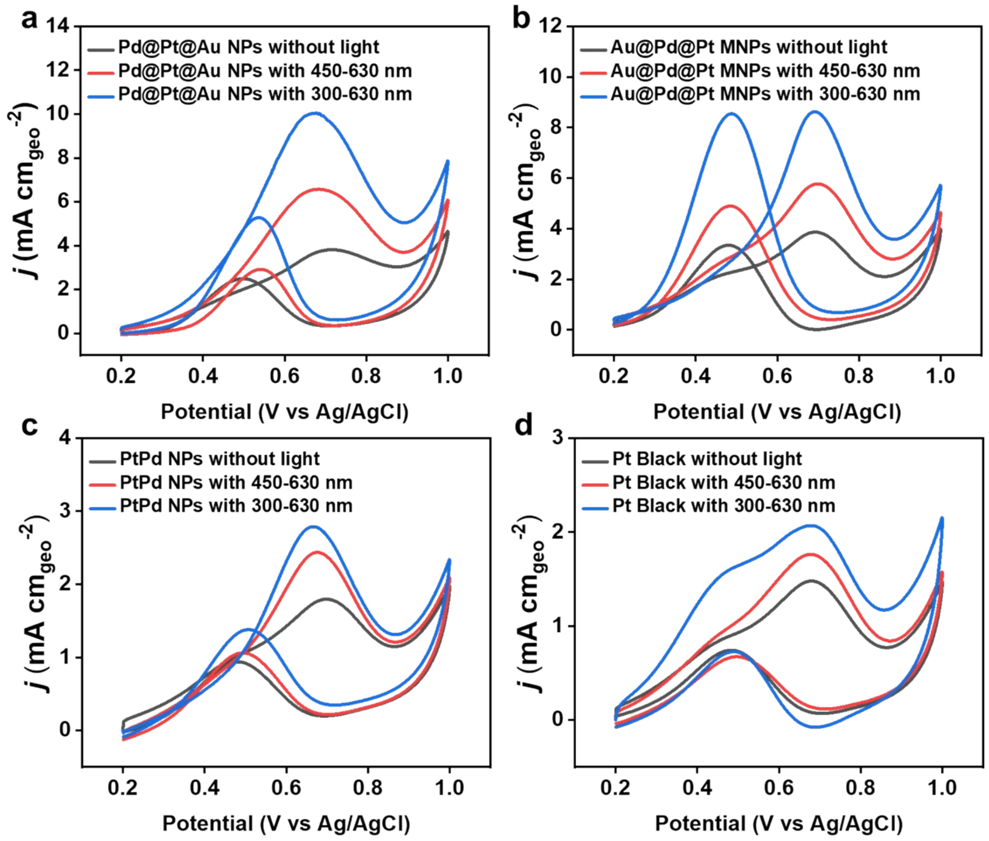

Figure S13. Geometrical area-normalized CV curves for methanol oxidation catalyzed by (a) Pd@Pt@Au MNPs, (b) Au@Pd@Pt MNPs, (c) Pd@Pt MNPs and (d) PtB respctively in 0.5M H_2SO_4 containing 0.5M methanol in different range of wavelength. All the CV curves were abtained at a scan rate of 50 mV s^{-1}.

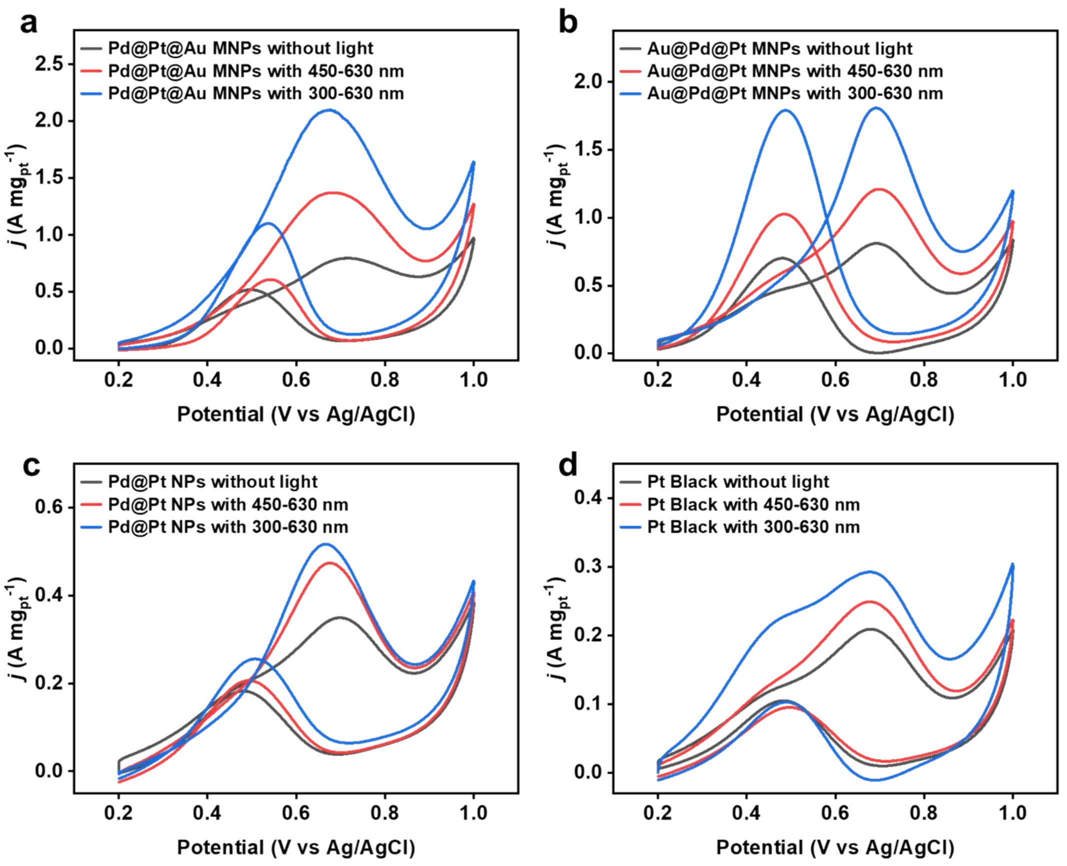

Figure S14. Mass-normalized CV curves for methanol oxidation catalyzed by (a) Pd@Pt@Au MNPs, (b) Au@Pd@Pt MNPs, (c) Pd@Pt MNPs and (d) PtB respctively in 0.5M H_2SO_4 containing 0.5M methanol in different range of wavelength. All the CV curves were abtained at a scan rate of 50 mV s^{-1}.

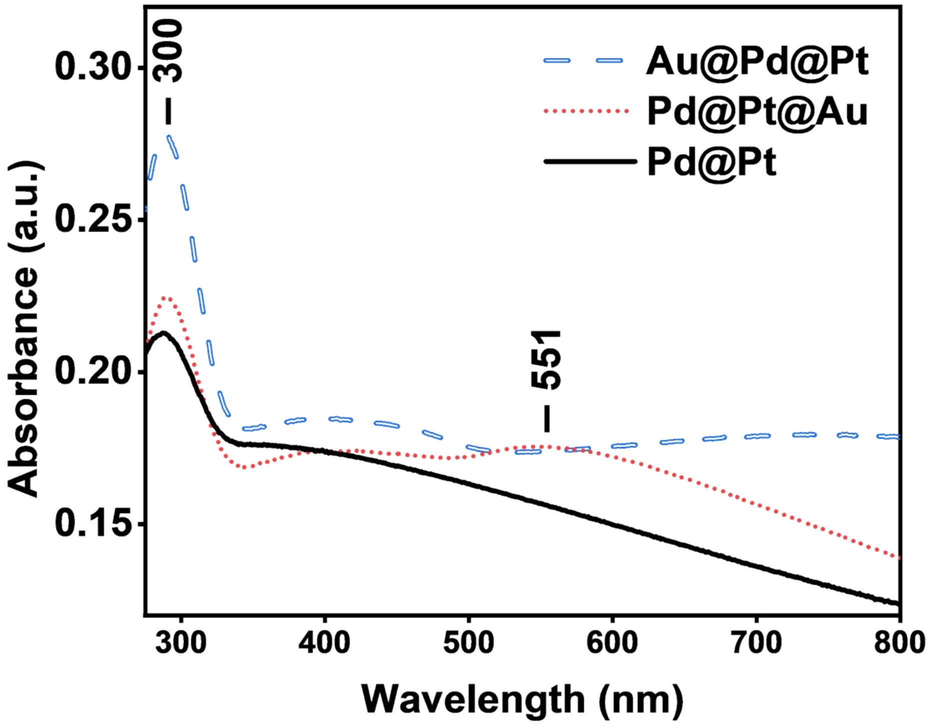

Figure S15. The absorbance spectrum of Pd@Pt@Au MNPs, Au@Pd@Pt MNPs, and Pd@Pt MNPs in the UV-Visible range.

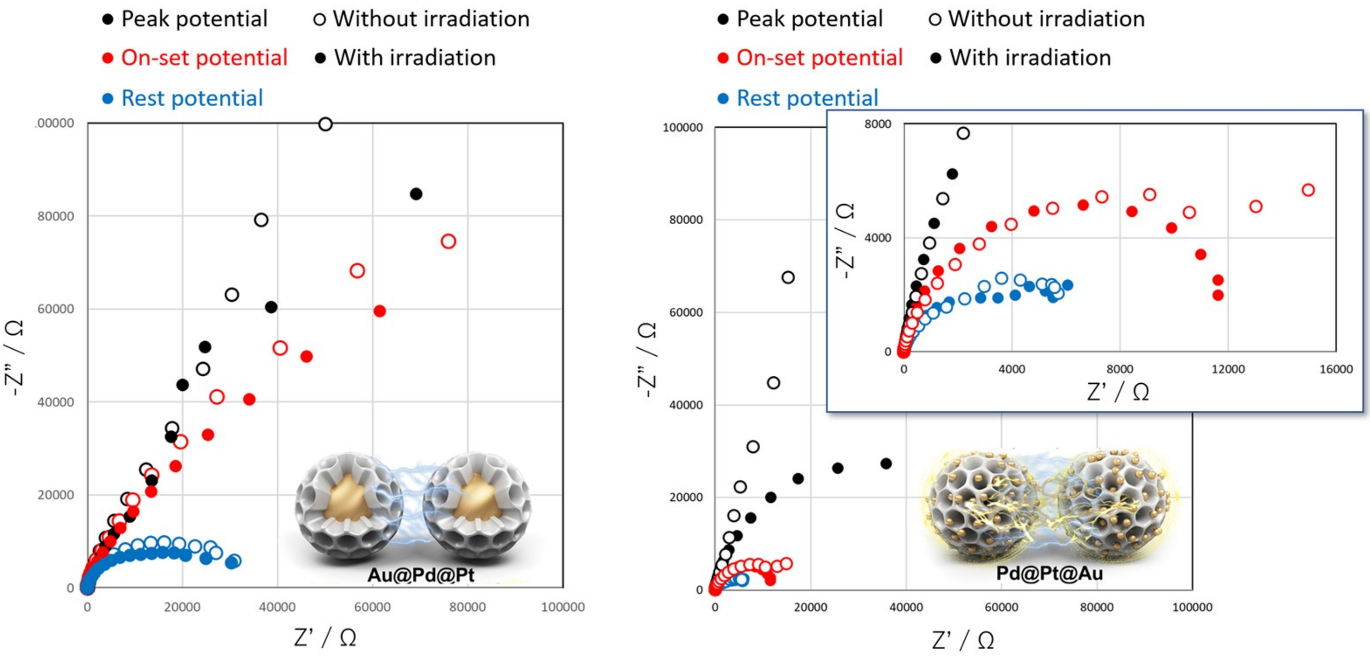

Figure S16. Nyquist plots of MOR for Pd@Pt@Au and Au@Pd@Pt MNPs under laser irradiation and non-irradiated conditions. Nyquist plots were abtained at rest potential, on-set potential (potential at 0.1 mA mg_{Pt}^{-1}), and peak current potential.

Note for Figure S16:

At the open-circuit potential (OCP), semicircles are observed in the Nyquist plots of both Pd@Pt@Au and Au@Pd@Pt MNPs. However, the impedance response of Pd@Pt@Au MNPs is significantly smaller than that of Au@Pd@Pt MNPs, indicating that the catalytic performance is influenced by the positioning of Pt and Pd. This result suggests that Pd@Pt@Au exhibits higher activity for MOR because Pt, with its high surface area, is located on the particle surface, as confirmed by element mapping. Although laser irradiation causes a slight reduction in impedance, no significant difference is observed.

At the onset potential, where both current density and overpotential are low, the Nyquist plots reveal a clear distinction between the two MNPs. These responses reflect dynamic EIS, meaning the MOR reaction can only be interpreted qualitatively. Pd@Pt@Au MNPs consistently exhibit smaller impedance compared to Au@Pd@Pt MNPs. In the Nyquist plots, semicircles are observed for Pd@Pt@Au MNPs, whereas Au@Pd@Pt MNPs display linear impedance behavior in the low-frequency region, characteristic of diffusion resistance. This suggests that MOR on Pd@Pt@Au MNPs follow typical diffusion-limited behavior under dynamic EIS conditions, while Au@Pd@Pt MPNs show static EIS behavior similar to its response at open-circuit potential. The superior methanol diffusion for Pd@Pt@Au MNPs can be attributed to the placement of Pt on the particle surface, ensuring more accessible active sites. Although laser irradiation causes a slight reduction in both impedance responses, no significant change is observed.

At the peak potential, where the current density reaches its maximum and the overpotential is sufficient for the reaction to proceed efficiently, the Nyquist plots reveal a significant difference between the two MNPs. These impedance responses also represent dynamic impedance, associated with high current density, and thus provide only qualitative insight. Neither Nyquist plot of MNPs show semicircles, with both showing linear

impedance behavior at low frequencies, indicating diffusion resistance under non-irradiated conditions. Under laser irradiation, however, the impedance responses change significantly at this potential. For Au@Pd@Pt MNPs, the slope of the linear impedance response decreased slightly, suggesting a small effect on the MOR mechanism from laser excitation. In contrast, Pd@Pt@Au MNPs exhibit a shift from linear to semicircular impedance behavior under laser irradiation. This change suggests a substantial reduction in diffusion impedance and a decrease in reaction resistance, although the precise cause, whether purely from the reaction or a combination of reaction and diffusion, remains uncertain.

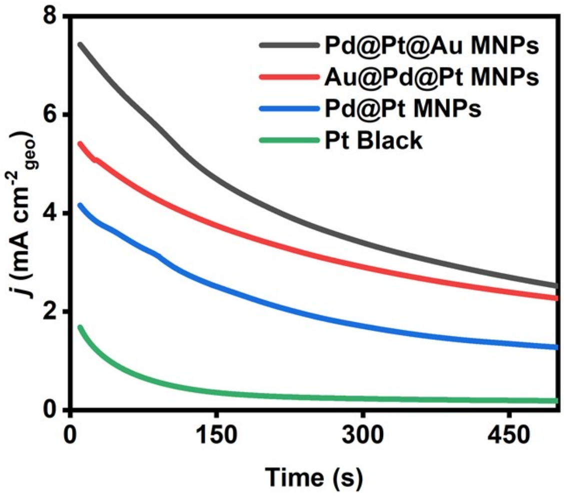

Figure S17 Stability test for MOR catalyzed by Pd@Pt@Au MNPs, Au@Pd@Pt MNPs, Pd@Pt MNPs, and PtB, respectively.

Supplementary Table S1. The standard reduction potential of different half reactions for various metal precursors.

Half reaction	E^0 (V *vs.* SHE)
$[PdCl_4]^{2-} + 2e^- \rightarrow Pd(s) + 4Cl^-$	+0.64
$[PtCl_6]^{2-} + 2e^- \rightarrow [PtCl_4]^{2-} + 2Cl^-$	+0.726
$[PtCl_4]^{2-} + 2e^- \rightarrow Pt(s) + 4Cl^-$	+0.758
$[AuCl_4]^- + 3e^- \rightarrow Au(s) + 4Cl^-$	+1.002

Supplementary Table S2. The ICP–OES analysis of Pd@Pt@Au MNPs and Au@Pd@Pt MNPs.

Sample	Atomic content of the elements (at%)		
	Pt	Pd	Au
Pd@Pt@Au MNPs	67.4	7.1	25.5
Au@Pd@Pt MNPs	67.6	7.5	24.9

Supplementary Table S3. The MOR performance of recently reported MNPs electrocatalysis.

**Catalysts**	**Mass activity (A mg_{Pt}^{-1})**	**Electrolyte**	**Reference**
Pd@Pt@Au MNPs - light	2.10	0.5 M H_2SO_4 0.5 M CH_3OH	This work
Au@Pd@Pt MNPs - Light	1.81		
Pd@Pt@Au MNPs	0.80		
Au@Pd@Pt MNPs	0.81		
PtPdCu Spheres	0.43	0.5 M H_2SO_4 0.5 M CH_3OH	1
Au@PdPt spheres	0.42		2
PtCu MNs	0.31		3
Au-PtNi DNPs	0.53		4
m-Pd/PdPt film	0.54		5
Pt@mPtRu YSs	0.56	0.5 M H_2SO_4 1.0 M CH_3OH	6
PtPdRuTe NTs	1.26		7
PtPdCu NDs	0.69		8

Supplementary References

(1) Jiang, B.; Li, C.; Malgras, V.; Yamauchi, Y. Synthesis of ternary PtPdCu spheres with three-dimensional nanoporous architectures toward superior electrocatalysts. *Journal of Materials Chemistry A* **2015**, *3* (35), 18053-18058.

(2) Jiang, B.; Li, C.; Imura, M.; Tang, J.; Yamauchi, Y. Multimetallic Mesoporous Spheres Through Surfactant-Directed Synthesis. *Adv Sci (Weinh)* **2015**, *2* (8), 1500112.

(3) Kang, Y.; Jiang, B.; Alothman, Z. A.; Badjah, A. Y.; Naushad, M.; Habila, M.; Wabaidur, S.; Henzie, J.; Li, H.; Yamauchi, Y. Mesoporous PtCu Alloy Nanoparticles with Tunable Compositions and Particles Sizes Using Diblock Copolymer Micelle Templates. *Chemistry* **2019**, *25* (1), 343-348.

(4) Wang, S.; Ma, L.; Song, D.; Yang, S. Au Doping PtNi Nanodendrites for Enhanced Electrocatalytic Methanol Oxidation Reaction. *Nanomaterials (Basel)* **2023**, *13* (21).

(5) Jiang, B.; Li, C.; Qian, H.; Hossain, M. S. A.; Malgras, V.; Yamauchi, Y. Layer-by-Layer Motif Architectures: Programmed Electrochemical Syntheses of Multilayer Mesoporous Metallic Films with Uniformly Sized Pores. *Angew Chem Int Ed Engl* **2017**, *56* (27), 7836-7841.

(6) Yin, S.; Kumar, R. D.; Yu, H.; Li, C.; Wang, Z.; Xu, Y.; Li, X.; Wang, L.; Wang, H. Pt@Mesoporous PtRu Yolk–Shell Nanostructured Electrocatalyst for Methanol Oxidation Reaction. *ACS Sustainable Chemistry & Engineering* **2019**, *7* (17), 14867-14873.

(7) Ma, S. Y.; Li, H. H.; Hu, B. C.; Cheng, X.; Fu, Q. Q.; Yu, S. H. Synthesis of Low Pt-Based Quaternary PtPdRuTe Nanotubes with Optimized Incorporation of Pd for Enhanced Electrocatalytic Activity. *J Am Chem Soc* **2017**, *139* (16), 5890-5895.

(8) Chang, R.; Zheng, L.; Wang, C.; Yang, D.; Zhang, G.; Sun, S. Synthesis of hierarchical platinum-palladium-copper nanodendrites for efficient methanol oxidation. *Applied Catalysis B: Environmental* **2017**, *211*, 205-211.
